# A novel gene signature combination improves the prediction of overall survival in urinary bladder cancer

**DOI:** 10.7150/jca.30307

**Published:** 2019-10-03

**Authors:** Siteng Chen, Ning Zhang, Jialiang Shao, Tao Wang, Xiang Wang

**Affiliations:** 1 Department of Urology, Shanghai General Hospital, Shanghai Jiao Tong University School of Medicine, Shanghai, China; 2 Department of Urology, Ruijin Hospital, Shanghai Jiao Tong University School of Medicine, Shanghai, China

**Keywords:** Urinary bladder cancer, Gene signature, Overall Survival, GSEA, TCGA

## Abstract

**Objectives**: Bladder carcinoma is a clinical heterogeneous disease, which is with significant variability of the prognosis and high risk of death. This revealed prominently the need to identify high-efficiency cancer characteristics to predict clinical prognosis.

**Methods**: Gene expression profiles of 93 bladder tumor patients from Gene Expression Omnibus data sets was performed in this study, along with 408 bladder tumor patients retrieved from The Cancer Genome Atlas database. The relationship of gene signature and overall survival was analyzed in the training cohort (n = 46). The validation for that was performed in an internal validation cohort (n = 47) and an external validation cohort (n = 408).

**Results**: Four genes (TMPRSS11E, SCEL, KRT78, TMEM185A) were identified by univariable and multivariable Cox regression analysis. According to a risk score on the bases on the four-gene signature, we grouped these patients in high-risk group and low-risk group with significantly different overall survival in the training series and successfully validated it in both the internal and external validation cohorts. Subsequent studies demonstrated that the four-gene expression risk score was independent of radical cystectomy stage, chemotherapy and lymph node status. Higher rates of *FAT4* mutation and *MACF1* mutation in bladder tumors with high risk score were found compared with tumors with low risk score. Gene set enrichment analysis revealed high-risk score was associated with some tumor progression and recurrence associated pathways.

**Conclusions**: This four-gene risk score might have potential clinical implications in the selection of high-risk urinary bladder cancer patients for aggressive therapy. The selected four genes might become potential therapeutic targets and diagnostic markers for urinary bladder cancer.

## Introduction

As the seventh most widely diagnosed carcinoma in the male population around the world 1, urinary bladder cancer has become one of the main death causes of Urologic cancer. And in 2017, approximately 81,190 new cancer cases and 17,240 cancer deaths related to urinary bladder cancer are estimated to occur in United States 2. Muscle-invasive urinary bladder cancer (MIBC) makes up for about 25% of initially diagnosed bladder carcinoma cases, whereas up to 10% to 15% of patients with non-muscle-invasive urinary bladder cancer (NMIBC) will progress to MIBC 3, 4. More importantly, approximately 5% to 15% of urinary bladder cancer patients are together with metastatic disease at the time of initial diagnosis 5. Accurate prediction of tumor progression and survival is important to determine the appropriate diagnosis and therapy decision. Thus, there is a clear need for predictive and prognostic markers that can identify tumor characteristics and predict clinical behavior.

Recently, gene signature has been used widely for risk stratification of patients with cancer 6. Many urinary bladder cancer gene signatures are reported to classify urinary bladder cancer patients into groups with distinct clinical outcomes 7-9. However, most current gene signatures for urinary bladder cancer relate to disease diagnosis only, with limited information about the prognostic value estimation, which will limit the clinical application of these signatures.

This current study is based on publicly available data sets, resulting in developing special genetic biomarkers associated with urinary bladder cancer survival closely, and evaluating the predictive value of this gene expression signature among urinary bladder cancer patients.

## Materials and Methods

### Gene profiles and microarray data

Raw CEL data and corresponding clinical data of microarray data from GSE31684 data sets 10, which used the Human Genome U133 Plus 2.0 chips platform of Affymetrix, were retrieved from Gene Expression Omnibus (GEO) database and then background adjusted by Robust Multichip Average 11. Gene profiles and clinical data for bladder tumors were downloaded from The Cancer Genome Atlas (TCGA) data base (March 2018). Patient selection was restricted to the urinary bladder cancer patients with gene expression data and essential clinical data, including clinical outcome and staging information. Patients without clinical survival information were removed from the study. In addition, patients with an overall survival (OS) of less than 1 month were also eliminated owing to possible unrelated causes of death. Finally, 93 patients from GSE31684 series and 408 patients from TCGA database were included in our study. The urinary bladder cancer samples of GSE31684 series were stochastic grouped into a training cohort (n = 46) or an internal validation cohort (n = 47). Additionally, urinary bladder cancer samples in TCGA database were analyzed as an external validation cohort. Mutation annotation format (MAF) of the TCGA cohort was also analyzed by the package of “maftools” to summarize, analyze, annotate and visualize MAF files in an efficient manner 12. The demographic, clinical and pathological information of bladder cancer patients from the GSE31684 and TCGA cohorts were shown in Table 1. The work has been reported in line with the REMARK criteria.

### Construction of risk score

**Fig 1:**
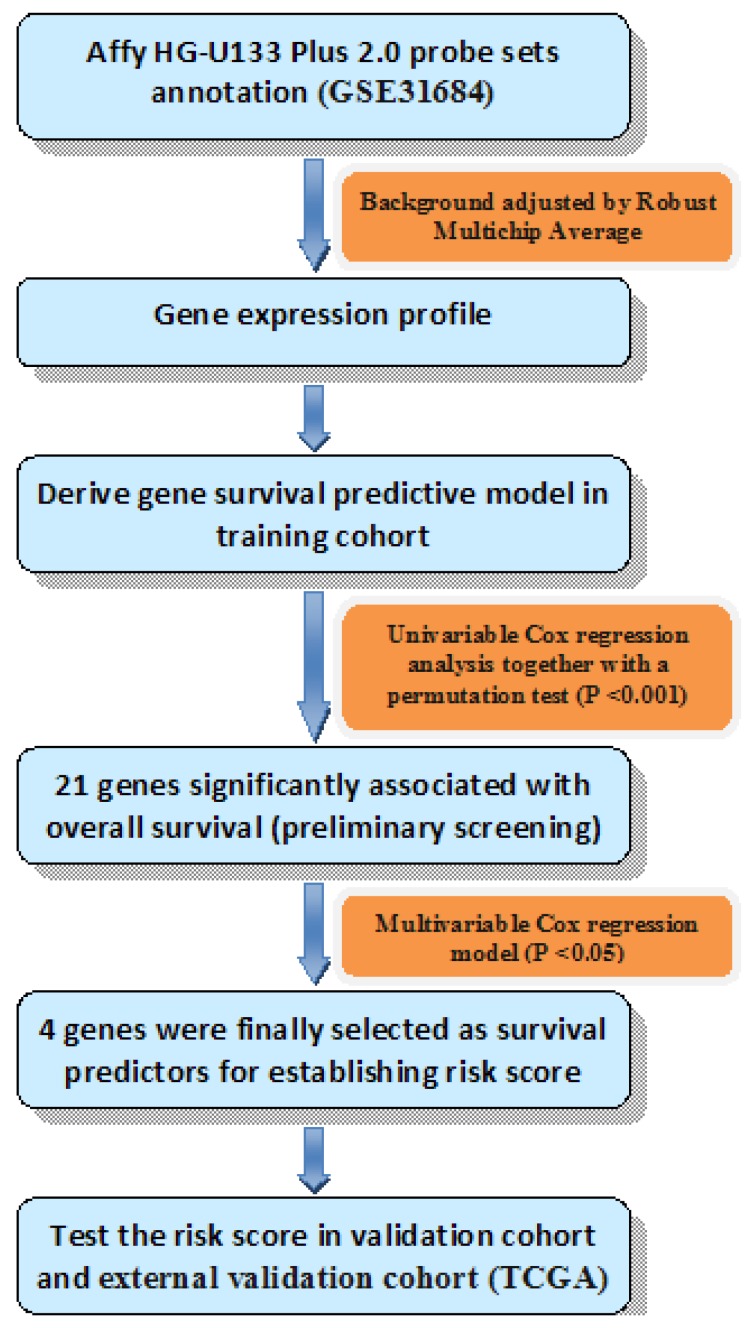
Diagram of the construction of risk score. The order of analyses to develop the risk score model and validate the efficiency of the gene signature to predict overall survival.

Univariable Cox regression analysis together with a permutation test 13 was used to explore the relationship of gene expression and OS. Genes would be considered with strong correlation with survival if they had a permutation *p* value less than 0.001 14, and then analyzed in the training series by multivariable Cox regression analysis. Genes were finally selected if their *p* values were less than 0.05 in the multivariable Cox analysis. Thus, we could establish the risk score formula by weighting multivariable regression coefficients of each selected gene. Patients were split into high risk group and low risk group using the median risk score of each series as a cutoff point. Diagram of the construction of risk score was shown in Fig. 1.

We performed a data stratification analysis and multivariable Cox regression analysis to verify the prediction power of risk score in bladder carcinoma patients. Receiver operating characteristic (ROC) curves and area under the curve (AUC) were calculated to contrast the specificity and sensitivity of OS status predictions at follow-up on the bases of genetic risk score, radical cystectomy (RC) stages and lymph node status.

### Gene Set Enrichment analysis

Gene Set Enrichment Analysis (GSEA) was set up in TCGA series using a molecular signatures database (MSigDB) C2 CP: Canonical pathway gene set collection 15. The GSEA, which was visualized in Enrichment Map software and Cytoscape 16, was applied to determine if the members of a given gene set were strikingly associated with our risk score. We carried out random sample permutations of 1000. The significance threshold was set at false discovery rate (FDR) < 0.01.

### Statistical analysis

Kaplan-Meier estimate was used for comparing survival differences between the low-risk group and the high-risk group. Hazard ratio (HR) with a 95% confidence interval (*CI*) was used in Cox regression analysis. And a P value less than 0.05 was considered significant. All the data were analyzed by R program 3.3.2 (www.rproject.org) and SPSS 13.0 (SPSS Inc., Chicago, IL, USA).

## Results

### Identification of prognostic genes and risk score formula in the training cohort

Firstly, we discovered a series of twenty-one genes with a parametric *P* value less than 0.001 by univariable Cox regression analysis. These twenty-one genes were further analyzed by applying multivariable Cox regression analysis in the training cohort. With this method, only four genes with *P* value less than 0.05 were selected as the predictors (Table 2).

Next, the risk score formula was established on the bases of the 4-gene expressions for OS prediction, as follows: risk score = (1.414*expression level of TMPRSS11E) + (2.471*expression level of SCEL) + (5.305*expression level of KRT78) + (-2.988* expression level of TMEM185A).

### The relationship between four-gene signature risk score and OS in the training series

**Fig 2:**
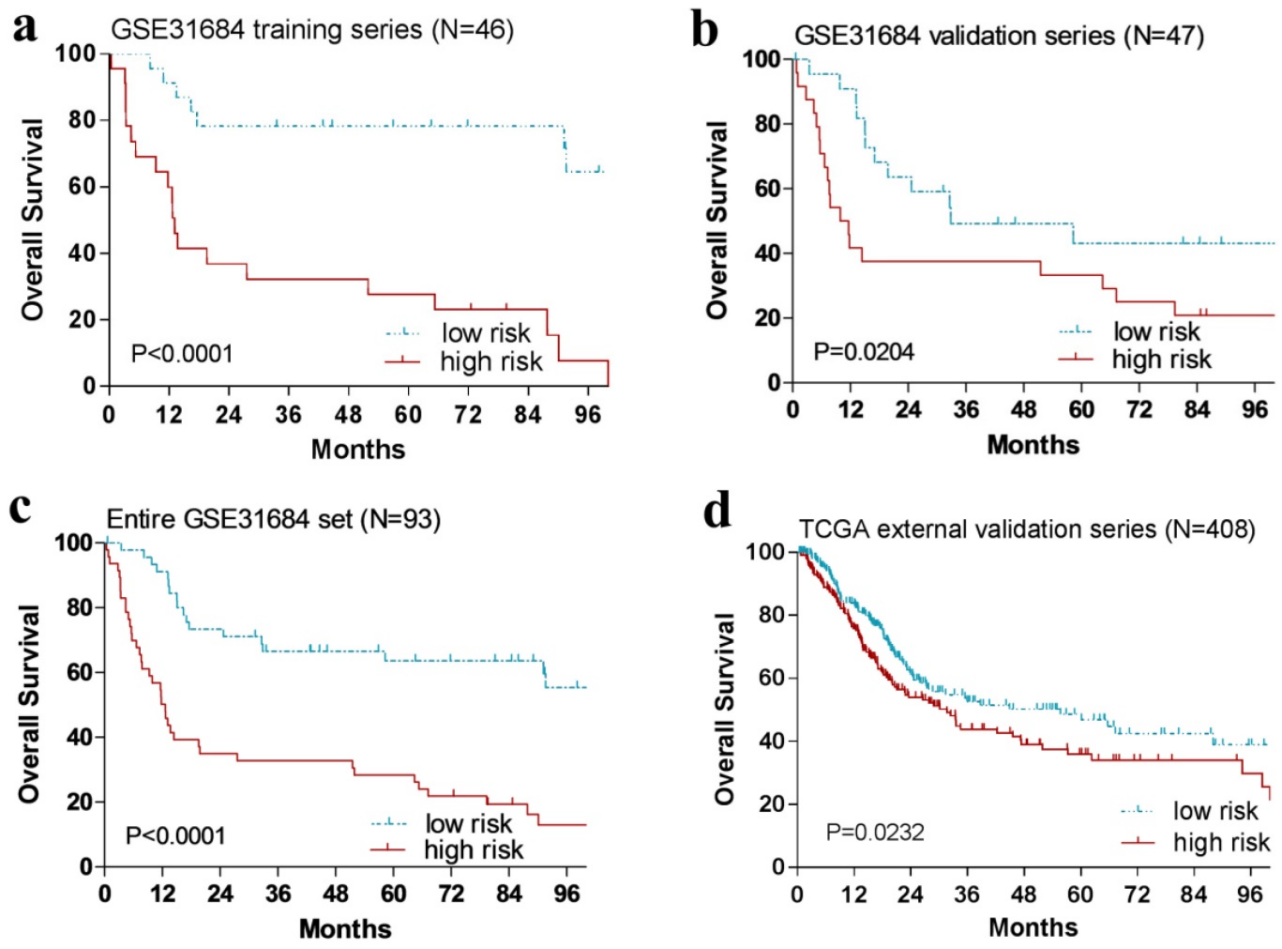
Kaplan-Meier estimates of the overall survival (OS) of urinary bladder cancer patients using the four-gene signature. The Kaplan-Meier plots were used to visualize the OS probabilities for the low-risk versus high-risk group of patients based on the median risk score from corresponding datasets patents. (**a**)Kaplan-Meier curves for GSE31684 training series patients (N = 46); (**b**) Kaplan-Meier curves for GSE31684 validation series patients (N = 47); (**c**) Kaplan-Meier curves for the entire GSE31684 set patients (N = 93). (**d**) Kaplan-Meier curves for the external TCGA validation series patients (N = 408). The tick marks on the Kaplan-Meier curves represent the censored subjects. The differences between the two curves were determined by the two-side log-rank test.

With the risk score formula above, the patients in the training cohort (n = 46) were grouped into a high-risk group (n = 23) or a low-risk group (n = 23) by the median risk score. And patients with high-risk score had remarkably shorter OS than those in the low-risk group (*P* < 0.0001, Fig. 2a).

### Validation of this four-gene signature risk score for OS prediction in the internal and external validation series

In order to confirm our findings, we repeated the survival analysis in the internal validation cohort (n = 47). Similarly, patients with a high risk score had a notable shorter OS than that in the low-risk group (*P* = 0.0204, Fig. 2b). We further validated our four-gene signature in the external TCGA validation cohort (n = 408). Specifically, patients could also be segregated into a low-risk group and a high-risk group by the median risk score of TCGA cohort (*P* = 0.0232, Fig. 2d).

### The predictive accuracy of the four-gene signature risk score compared with other clinical factors

The Cox regression analysis revealed that our four-gene risk score was strikingly associated with survival as a continuous variable in the entire GSE31684 cohort and TCGA cohort (Table 3). In the entire GSE31684 dataset, only the risk score, muscle invasion and lymph node status were remarkably associated with OS. Next, we carried out multivariable Cox regression proportional hazards regression analysis to explore whether our four-gene risk score could act as an independent survival predictor. Similar results of Cox regression analysis were also observed in the TCGA cohort. These results demonstrated that our risk score was still significantly associated with the OS when adjusted by age at diagnosis, muscle invasion, lymph node status and chemotherapy, which suggests that the four-gene signature risk score could serve as an independent survival predictor for urinary bladder cancer.

### Assessment of the risk prediction model

**Fig 3:**
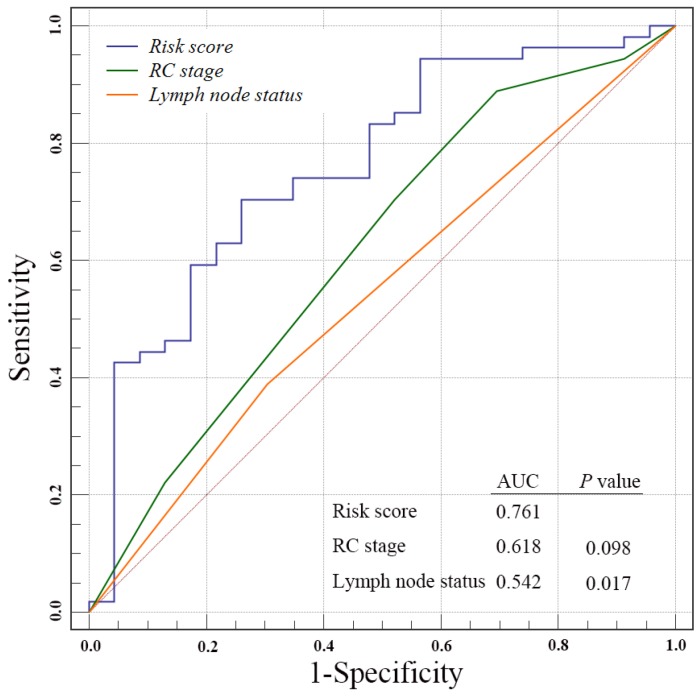
Receiver operating characteristic(ROC) analysis of the sensitivity and specificity of the overall survival status prediction at ten-year follow-up by the four-gene signature risk score, radical cystectomy (RC) stage and lymph nods status in the entire GSE31684 data set patients. P values were from the comparisons of the area under the curve (AUC) of four-gene signature risk score versus those of RC stage and lymph nods status respectively. The predictive ability of risk score was equivalent to RC stage (P = 0.098), but better than lymph nods status (P = 0.017)

We next to perform ROC analysis to evaluate the specificity and sensitivity of the OS status prediction at ten-year follow-up by the four-gene signature risk score, RC stage and lymph node status in the entire GSE31684 data set patients. Notably, we found a better AUC of four-gene signature score compared with RC stages, even though without significant difference (0.761 versus 0.618, *P* = 0.098, Fig. 3). However, our risk score was strikingly superior to lymph node status (0.761 versus 0.542, *P* = 0.017).

### The prognostic risk score is independent of RC stages and postoperative chemotherapy

**Fig 4:**
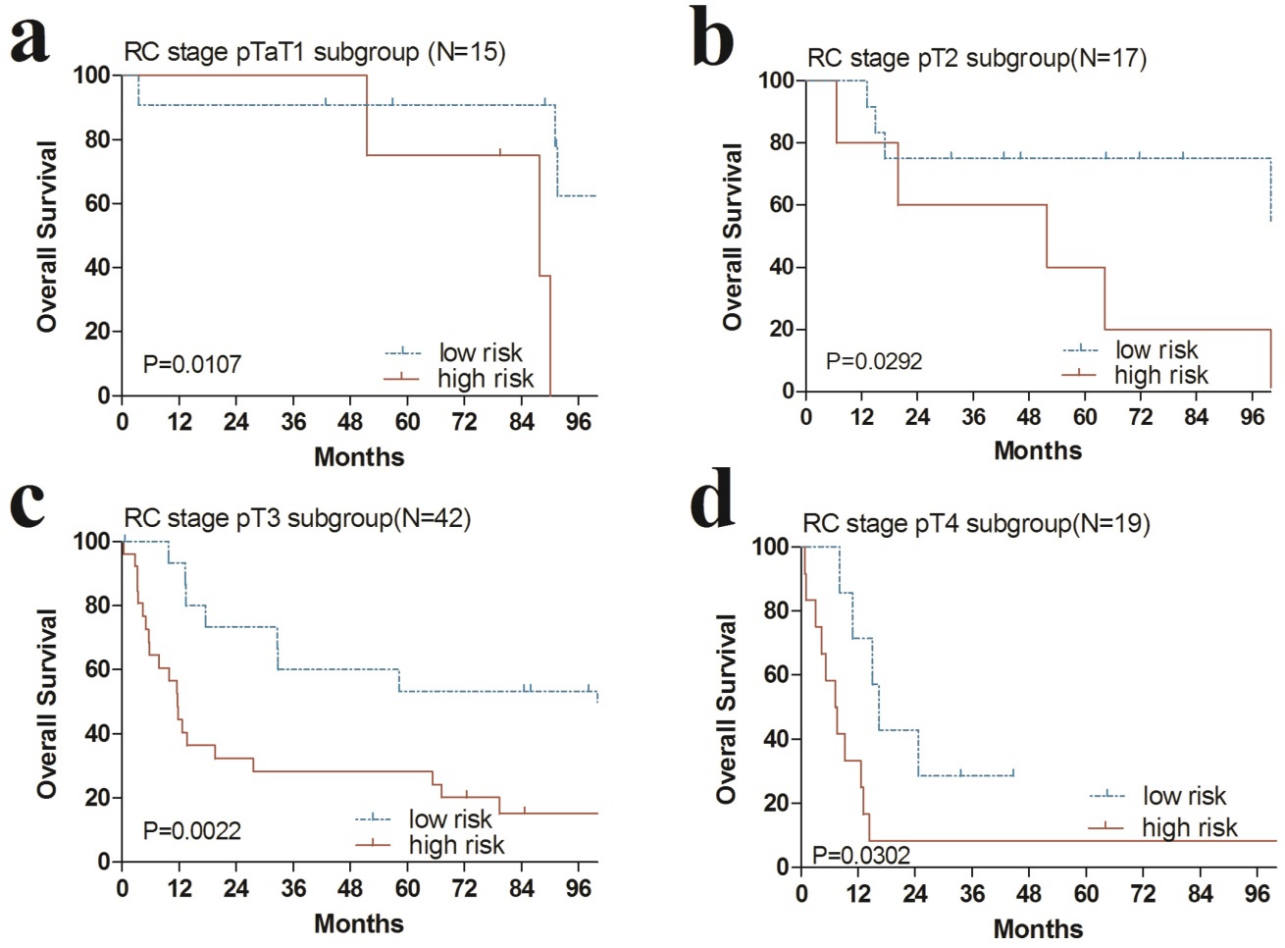
Kaplan-Meier estimates of the overall survival (OS) of GEO patients using the four-gene signature, stratified by radical cystectomy (RC) stage (pT1, pT2, pT3 & pT4). Kaplan-Meier plots were then used to visualize the survival probabilities for the high-risk versus low-risk group of patients determined on the basis of the median risk score from the entire GSE31684 set patients within each RC stage. (**a**) Kaplan-Meier curves for patients with RC stage pT1 (N=15); (**b**) Kaplan-Meier curves for patients with RC stage pT2 (N=17); (**c**) Kaplan-Meier curves for patients with RC stage pT3 (N=42); (**d**) Kaplan-Meier curves for patients with RC stage pT4 (N=19). The tick marks on the Kaplan-Meier curves represent the censored subjects. The differences between the two curves were determined by the two-sided log-rank test.

**Fig 5:**
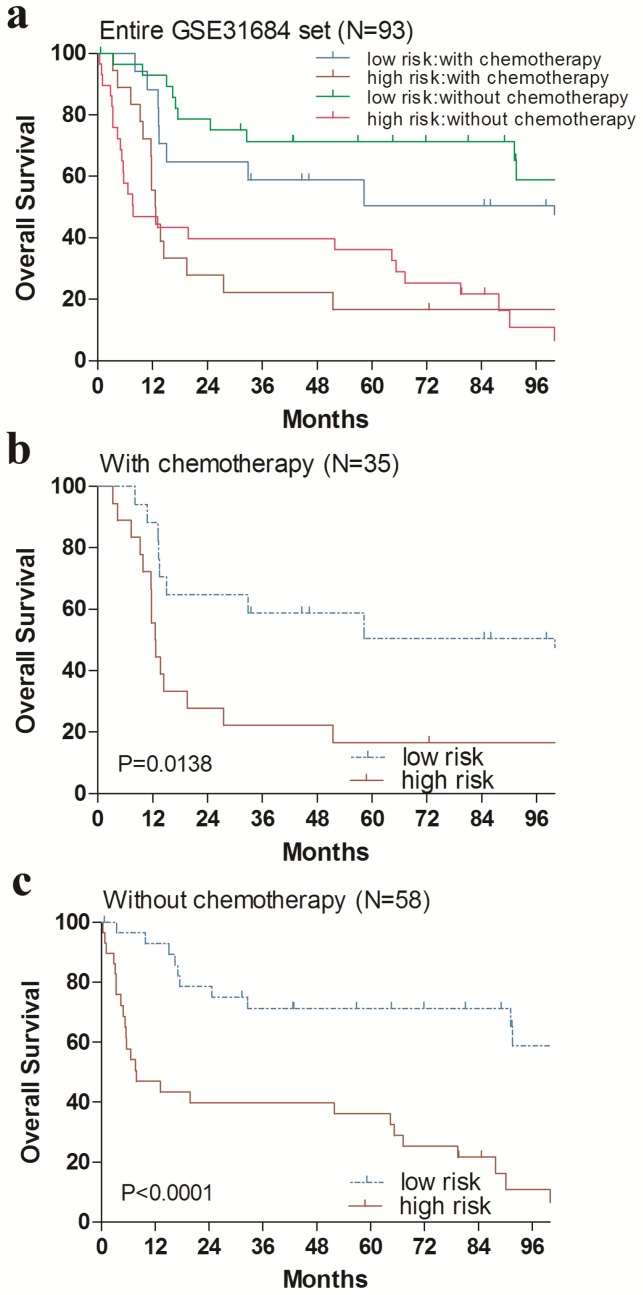
Kaplan-Meier estimates of the overall survival (OS) of GEO patients using the four-gene signature risk score, stratified by postoperative chemotherapy. Entire GSE31684 data set (N=93) were first stratified by postoperative chemotherapy (with or without postoperative chemotherapy). Kaplan-Meier plots were then used to visualize the survival probabilities for the high-risk versus low-risk group of patients determined on the basis of the median risk score from the entire GSE31684 data set patients within each postoperative chemotherapy stratum. (**a**) Kaplan-Meier curves for the entire GSE31684 data set patients (N = 93); (**b**) Kaplan-Meier curves for patients with postoperative chemotherapy (N=35); (**c**) Kaplan-Meier curves for patients without postoperative chemotherapy (N=58). The tick marks on the Kaplan-Meier curves represent the censored subjects. The differences between the two curves were determined by the two-sided log-rank test.

Additionally, we performed data stratification analysis to find whether the four-gene signature was independent of RC stage and chemotherapy. Our results revealed that this risk score also could divide urinary bladder cancer samples into those with longer survival and those with shorter survival in each RC stage subgroup (Fig. 4). Similarly, among those patients with or without chemotherapy, the four-gene signature risk score could also distinguish between patients with the significantly different OS (Fig. 5).

### Identification of four-gene risk score correlated biological pathways

**Fig 6:**
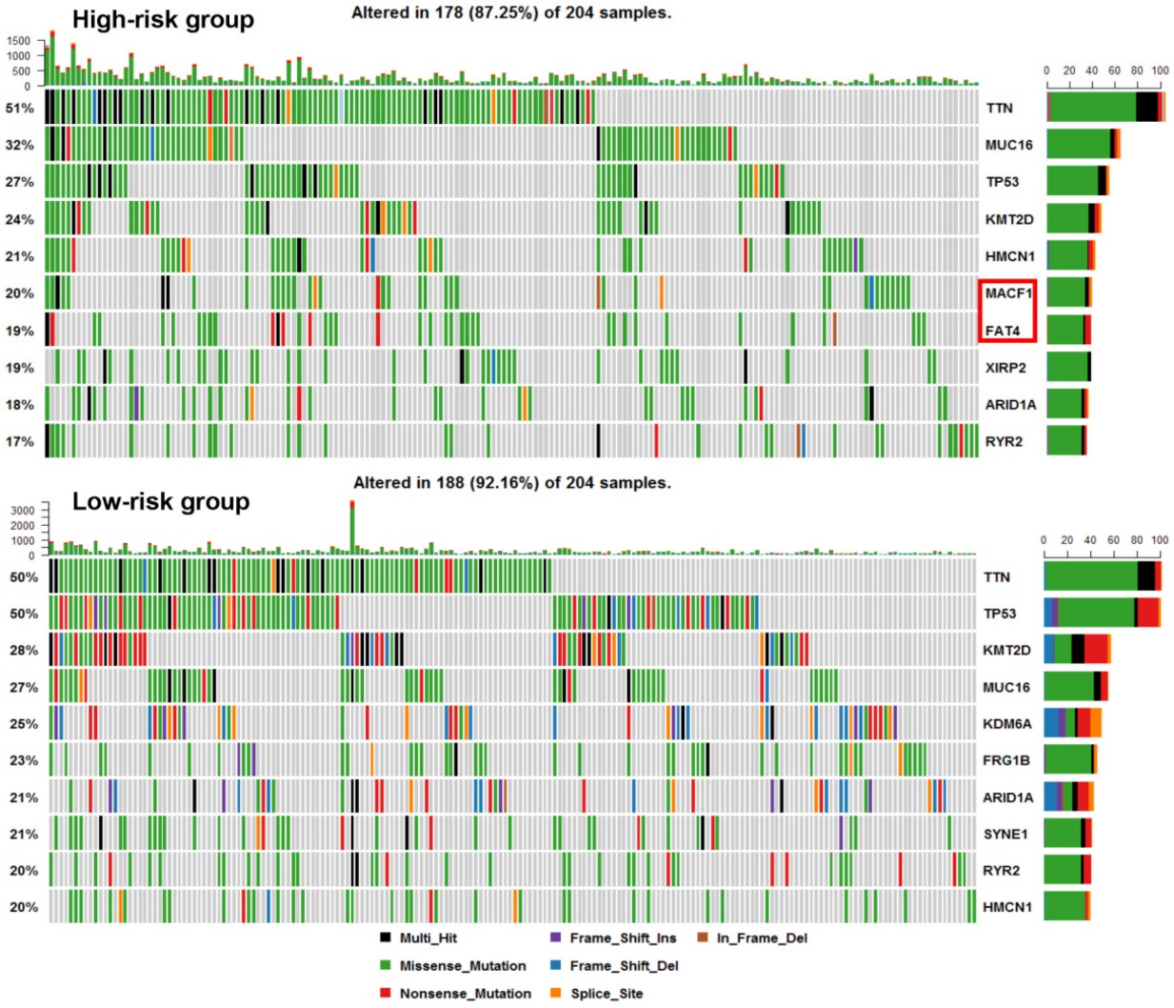
Alteration landscape for 204 bladder tumors with high risk score and 204 low-risk bladder tumors in TCGA cohort. Higher rates of *FAT4* mutation and *MACF1* mutation in bladder tumors with high risk score were found compared with tumors with low risk score.

**Fig 7:**
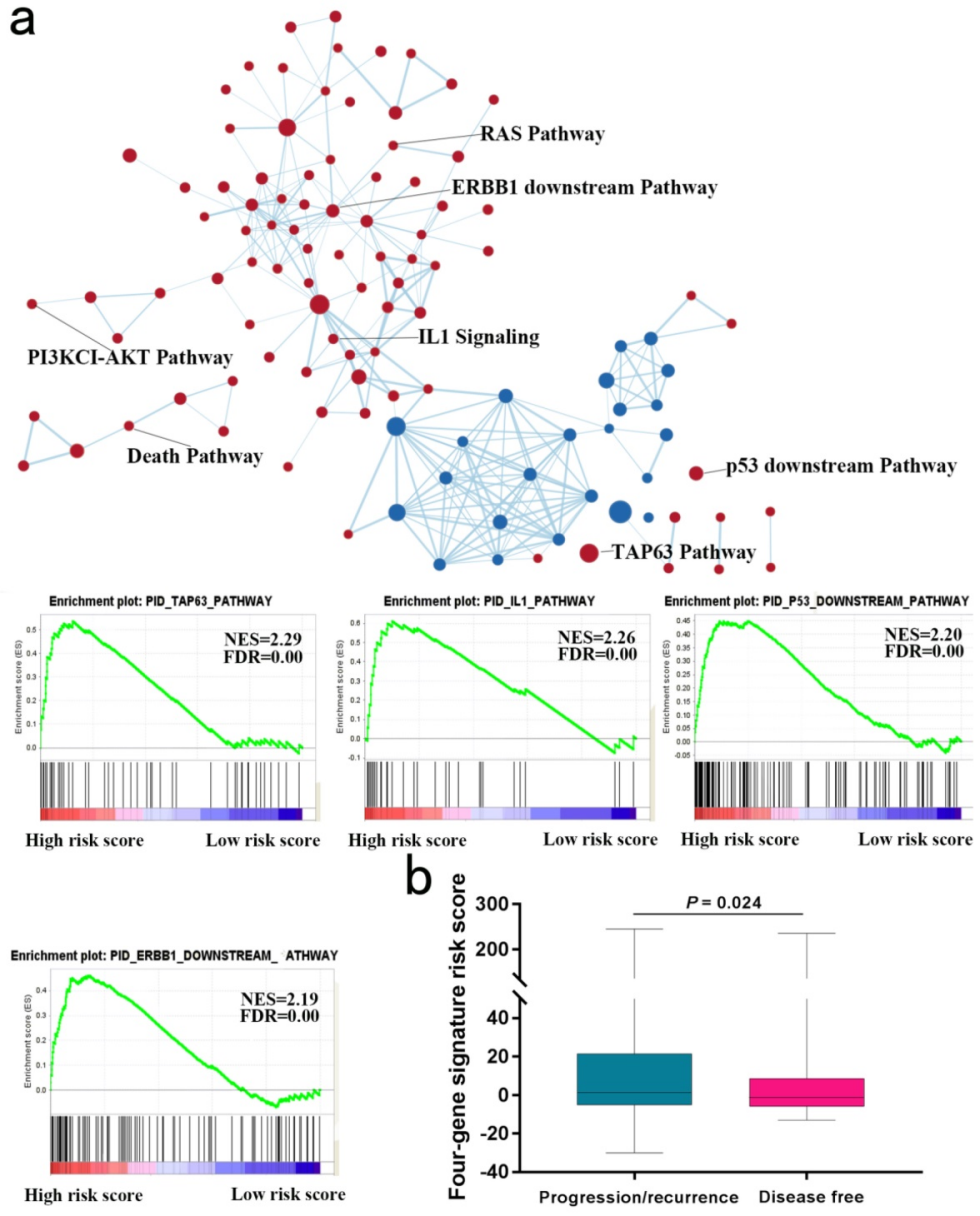
Gene Set Enrichment Analysis in TCGA database. (**a**) Gene Set Enrichment Analysis Delineates Biological Pathways and Processes associated with risk score. Cytoscape and Enrichment Map were used for visualization of the GSEA results. (**b**) Risk score of patients with or without progression/recurrence in TCGA series.

We next explored whether bladder tumors with high risk score were associated with specific tumor mutation. Alteration landscape bladder tumors with high or low risk scores were shown in Fig. 6. Eight genes were mutated in >19% of samples with high risk score: *TTN* (51%), *MUC16* (32%), *TP53* (27%), *KMT2D* (24%), *HMCN1* (21%), *MACF1* (20%), *FAT4* (19%) and *XIRP2* (19%). While ten genes were mutated in >19% of samples with low risk score: *TTN* (50%), *TP53* (50%), *KMT2D* (28%), *MUC16* (27%), *KDM6A* (25%), *FRG1B* (23%), *ARID1A* (21%), *SYNE1* (21%), *RYR2* (20%) and *HMCN1* (20%). Specifically, higher rates of *FAT4* mutation and *MACF1* mutation in bladder tumors with high risk score were found compared with tumors with low risk score. GSEA analysis revealed that high-risk score tended to be accompanied by a number of up-regulated networks including cancer progression and recurrence associated pathways (Fig. 7a). Bladder cancer patients with recurrence/progression tended to get a risk score higher than those with disease free (Fig. 7b).

## Discussion

Tumor stages and tumor grades are important predictors of tumor prognosis. Nevertheless, histopathological classifications tend to be limited by observer variability 17, 18. Recently urinary bladder cancer gene expression signatures have been generally applied to predict cancer characteristics and outcomes. For example, Smith SC 8 found a twenty-gene signature with statistically significant correlation with disease development among patients with urinary bladder cancer. Heijden 9 developed a five-gene expression signature to predict progression in T1G3 urinary bladder cancer. However, there are few relevant gene signatures reported to directly predict OS for patients with urinary bladder cancer. Here, we found a four-gene signature risk score remarkably associated with the OS of urinary bladder cancer, which was independent of RC stages and postoperative chemotherapy.

Our study has some novel approaches and findings. Firstly, compared to previous studies related to disease diagnosis only, our study focused on direct overall survival prediction of urinary bladder cancer patients, which were poorly reported before. Secondly, Cox regression analysis together with a permutation test (*P* value less than 0.001) and stratification analysis were performed to explore the relationship of gene expression and overall survival of bladder cancer. And thirdly, we firstly identified a set of 4 genes (*TMPRSS11E*, *SCEL*, *KRT78*, *TMEM185A*) that were significantly associated with poor overall survival of bladder cancer patients, some of which have not been investigated in urinary bladder cancer before. Moreover, we also revealed higher mutation rates of *FAT4* and *MACF1* in bladder tumors with higher risk score.

These four genes might become the potential therapeutic targets. They are worthy of further investigation to understand their role in the progression of bladder tumor. For example, the expression of TMEM185A (transmembrane protein 185A) was tended to be down-regulated clones among patients with stage III serous ovarian carcinoma 19. TMPRSS11E (transmembrane protease, serine 11E) could suppress esophageal squamous cell carcinoma development by sensitizing cells to apoptosis under an apoptotic stimulus through downregulating the EGFR/AKT signaling pathway 20, but recently was found significantly upregulated in urinary bladder cancer patients 21, which was consistent with our study outcomes.

Higher rates of *FAT4* mutation and *MACF1* mutation in bladder tumors with high risk score were found compared with tumors with low risk score. GSEA analysis revealed that high-risk score tended to be accompanied by a number of up-regulated networks including cancer progression and recurrence associated pathways. For instance, TAP63 pathway and IL1 pathway were implicated in cancer progression and recurrence 22, 23. Since tumor progression and recurrence are important risk factors for OS, we next to explore the difference of risk scores of samples with and without progression/recurrence by the TCGA cohort. Notably, patient with recurrence/progression tended to get a risk score higher than those with disease free.

By applying the four-gene risk scores to GSE31684 training cohort, a distinct separation was found in survival curves between patients with high-risk or low-risk signatures. The urinary bladder cancer patients with high-risk gene signatures tended to get shortened overall survival, while those with a low-risk signature tended to possess prolonged survival. The usefulness of the four-gene risk score could be validated in both the internal and external validation cohorts, indicating good reproducibility of our risk score for urinary bladder cancer patients.

Subsequent Cox proportional hazards regression analysis demonstrated that our predictive risk score was independent of some other importance prognostic clinical factors, including muscle invasive and lymph node status. Muscle-invasive disease is very important because the treatment and pathogenesis differ from MIBC to NMIBC 1, 24. As a result, it is of great importance to identify whether the prognostic value of this risk score is connected with the strong predictive clinical factor. Using multivariable Cox regression analysis, we identified that the prognostic value of our risk score was independent in MIBC patients.

Subsequently, stratification analysis suggested that the predictive value of the four-gene risk score was independent of RC stage and postoperative chemotherapy. An obvious OS benefit was found in a large retrospective cohort analysis in 3974 patients, with an HR of 0.75 for the high-risk subgroup with adjuvant chemotherapy 25. In this research, we observed that patients with urinary bladder cancer could be grouped into high-risk groups or low-risk groups by the four-gene risk score despite of chemotherapy stratum. Similarly, among those patients in each RC stage subgroup, the four-gene signature risk score could also distinguish between patients with the significantly different OS. All of this above strongly demonstrated that the four-gene signature risk score might serve as a remarkably prognostic factor for urinary bladder cancer.

The TNM staging system, which includes primary tumor stage, lymph node and distant metastasis, is the most accepted to be used to evaluate and predict the risk of tumor development and progression in clinical practice 26. In this study, the survival predictive value of the four-gene risk score was stronger than that of the RC stage in the ROC analysis(AUC: 0.761 versus 0.618), although without statistical significance (*P* = 0.098), but was significantly stronger than that of lymph node status (AUC: 0.761 versus 0.542, *P* = 0.017). These results demonstrated the potential prognostic power of our gene signature risk score in clinical.

Our research has some limitations. Firstly, we have not verified this risk score in a clinical trial. Secondly, the median risk score was used as a cutoff point for classifying as previous reports 14, 27, but a prior cutoff point should be found in further studies to more scientifically split patients in high-risk group or low-risk group. Finally, this bioinformatics analysis of the four-gene signature was not carried out in this study, and the biological roles of several genes in this signature were not clear, which should be investigated in further fundamental researches.

## Conclusion

In summary, we had identified a four-gene signature that was useful in overall survival prediction in urinary bladder cancer patients. The selected four genes might become potential therapeutic targets and diagnostic markers for urinary bladder cancer. Future studies will concentrate on functional approaches of the selected four genes and validation of our risk score in clinical trials.

## Figures and Tables

**Table 1:** Demographic, clinical and pathological information of the GSE31684 and TCGA cohorts.

	GSE31684			TCGA
	Total	Training cohort	Validation cohort	Validation cohort
**No. of patients**	93	46	47	408
**Age (year)**				
** Mean**	69.1	68.6	69.7	68.1
** Range**	41.7~91.1	47.5~85.1	41.7~91.1	34.0~90.0
**Gender**				
** Male**	68(73.1%)	33(71.7%)	35(74.5%)	301(73.8%)
** Female**	25(26.9%)	13(28.3%)	12(25.5%)	107(26.2%)
**Tumor stage**				
** Ta/T1**	15(16.1%)	9(19.6%)	6(12.8%)	4(1.0%)
** T2**	17(18.3%)	7(15.2%)	10(21.3%)	119(29.2%)
** T3**	42(45.2%)	20(43.5%)	22(46.8%)	194(47.5%)
** T4**	19(20.4%)	10(21.7%)	9(19.1%)	58(14.2%)
** Unknown**	0	0	0	33(8.1%)
**Tumor grade**				
** High**	6(6.5%)	42(91.3%)	45(95.7%)	384(94.1%)
** Low**	87(93.5%)	4(8.7%)	2(4.3%)	21(5.1%)
** Unknown**	0	0	0	3(0.8%)
**Lymph node status**				
** Positive**	28(30.1%)	13(28.3%)	15(31.9%)	129(31.6%)
** Negative**	49(52.7%)	25(54.3%)	24(51.1%)	237(58.1%)
** Unknown**	16(17.2%)	8(17.4%)	8(17.0%)	42(10.3%)
**Adjuvant therapy**	35(37.6%)	18(39.1%)	17(36.2%)	10(2.5%)
**Smoking packs/year**				
** Mean**	35.2	35	35.4	39
** Range**	0~120	0~120	0~120	0~730
**Informed consent (yes)**	93(100%)	46(100%)	47(100%)	408(100%)
**OS (month)**				
** Mean**	47.5	51.6	43.4	27
** Range**	0.39~175.5	0.39~175.5	0.66~173.4	0.43~168.3
**Vital status**				
** Living**	28(30.1%)	13(28.3%)	15(31.9%)	228(55.9%)
** Dead**	65(69.9%)	33(71.7%)	32(68.1%)	180(44.1%)
**Recurrence/progression**	39(41.9%)	20(43.5%)	19(40.4%)	141(34.6%)

Informed consent were accomplished by researchers of the GSE31684 and TCGA cohorts; TCGA, The Cancer Genome Atlas; OS, overall survival.

**Table 2:** Genes significantly associated with the overall survival in the training cohort (N= 46).

Gene symbol	RNA type	Chromosome location	Hazard ratio	Coefficient
TMPRSS11E	mRNA	4q13.2	2.199	1.414
SCEL	mRNA	13q22.3	1.661	2.471
KRT78	mRNA	12q13.13	11.98	5.305
TMEM185A	mRNA	Xq28	0.236	-2.988

**Table 3:** Univariable and multivariable Cox regression analyses in the GSE31684 and TCGA cohorts.

	Univarible model				Multivariable model		
Variables	HR	95%*CI*	*P* value		HR	95%*CI*	*P* value
**GSE31684 (N=96)**							
** Risk score**	1.104	1.063~1.147	<0.0001		1.072	1.025~1.121	0.002
** Age**	1.012	0.986~1.038	0.365		1.008	0.976~1.041	0.619
** Muscle invasive**	3.166	1.360~7.372	0.008		2.489	1.024~6.048	0.044
** Lymph node status**	2.346	1.298~4.241	0.005		2.057	1.049~4.035	0.036
** Chemotherapy**	1.158	0.698~1.921	0.57		0.661	0.350~1.250	0.203
**TCGA (N=408)**							
** Risk score**	1.005	1.002~1.008	<0.001		1.004	1.001~1.008	0.016
** Age**	1.032	1.017~1.048	<0.0001		1.033	1.016~1.050	<0.001
** Tumor stage (Ta~T2 vs T3~T4)**	2.115	1.461~3.062	<0.0001		1.671	1.113~2.508	0.013
** Lymph node status**	2.258	1.651~3.089	<0.0001		1.876	1.351~2.605	<0.001

HR, hazard ratio; *CI*, *confidence interval*; TCGA, The Cancer Genome Atlas.

## Abbreviations

MIBC: muscle-invasive urinary bladder cancer
NMIBC: non-muscle-invasive urinary bladder cancer
GEO: Gene Expression Omnibus
TCGA: The Cancer Genome Atlas
OS: overall survival
HR: hazard ratio
CI: confidence interval
RC: radical cystectomy
GSEA: Gene Set Enrichment Analysis
FDR: false discovery rate
ROC: receiver operating characteristic
AUC: curves and area under the curve
